# Relative Age Effects in Dutch Adolescents: Concurrent and Prospective Analyses

**DOI:** 10.1371/journal.pone.0128856

**Published:** 2015-06-15

**Authors:** Bertus F. Jeronimus, Nikolaos Stavrakakis, René Veenstra, Albertine J. Oldehinkel

**Affiliations:** 1 Interdisciplinary Center Psychopathology and Emotion regulation (ICPE), Department of Psychiatry, University Medical Centre Groningen, University of Groningen, Groningen, The Netherlands; 2 Department of Sociology and Interuniversity Center for Social Science Theory and Methodology (ICS), University of Groningen, Groningen, The Netherlands; University of Western Brittany, FRANCE

## Abstract

The literature on relative age position effects is rather inconsistent. In this study we examined intra-classroom age position (or relative age) effects on Dutch adolescents’ school progress and performance (as rated by teachers), physical development, temperamental development (fear and frustration), and depressive symptoms, all adjusted for age at the time of measurement. Data were derived from three waves of Tracking Adolescents' Individuals Lives Survey (TRAILS) of 2230 Dutch adolescents (baseline mean age 11.1, SD = 0.6, 51% girls). Albeit relative age predicted school progress (grade retention ORs = 0.83 for each month, skipped grade OR = 1.47, both *p*<.001), our key observation is the absence of substantial developmental differences as a result of relative age position in Dutch adolescents with a normative school trajectory, in contrast to most literature. For adolescents who had repeated a grade inverse relative age effects were observed, in terms of physical development and school performance, as well as on depressive symptoms, favoring the relatively young. Cross-cultural differences in relative age effect may be partly explained by the decision threshold for grade retention.

## Introduction to Relative Age

*“I can honestly say that insecurity was something formerly unknown to me*. *I was always the best of my grade*. *The tallest*, *the fastest–I thought I was Superman*. *It turned out this was mainly because I was born in January*, *thus older than my peers”*. Gert Verhulst, as interviewed by Sara Berkeljon for the Volkskrant, March 23, 2013.

In most countries, children at school are assorted in same-age groups based on the month and year of birth [1,2]. Consequently, within a single classroom children may differ in age by up to 11 months. Relatively older children have a slightly more developed physique and mind than their younger classmates [1,3]. These physical and psychological advantages may become catalyzed into different developmental trajectories through favorable peer-contrast effects [4,5]. We define developmental differences due to the age position driven peer contrast effects as relative-age effects.

Taller and more mature children tend to have more prestige [6,7], which in turn affects friendship formation [8,9], and enhances learning opportunities [10]. Relative-age has been associated with higher intelligence [11], school success [12], identity formation [13], peer-perceived competence and leadership [14], success in sports [15], and positive self-perception and self-esteem [16,17]. Children’s internal working models are based on self- and other representations (“looking glass self”), which emerge and crystallize relatively early in development [18,19]: five-year olds have already stable and clearly established classroom hierarchies [20].

### Self-fulfilling prophecy

Children’s relative-age position may become a self-fulfilling prophecy (“learning by being”), catalyzed by reciprocal feedback loops between the developing phenotypes and their environments [13,21]; analogous to the corresponsive principle in the temperament and personality literature [22,23] and the Dickens-Flynn model [24,25]. For example, more positive adult evaluations for relatively older children based on favorable intra-cohort contrast effects [1] may drive repetitious advantageous social interactions, which influences their subsequent development. Relatively old children are granted special opportunities for success, such as relatively higher grades and extra coaching in sports [4,15,26], whereas relatively young children meet lower expectations and have an increased risk to repeat a grade [3,12,27].

Though classroom hierarchies are established in childhood, their consequences may be particularly salient in adolescence [28], due to the accumulative nature of their effects (a “halo” *vs*. “pitchfork” effect [29]). Adolescents become increasingly able to influence their environment while parental socialization wanes, and select a rapidly expanding peer network, and a first romantic partner [30,31]. Furthermore, earlier work associated being relatively young with victimization [32], psychiatric problems [33,34], and suicide before age 20 [35]. Because unfavorable relative-age contrast effects modulate children’s self-perception and self-esteem [16], which are known risk factors for affective disorders [36], being relatively young may increase risk of depression, which has a high incidence in adolescence [37,38]. Because low self-perception and self-esteem can have a persistent impact on affect and temperament [39,40], a relative-age effect might also be discernible in the development of temperamental negative affect over puberty. This has never been tested.

Finally, relatively older children tend to play more sports [4,21], partly driven by selection effects, which may explain observed relative-age effects on multiple indices of physical growth in adolescence [41]. Timing of puberty onset seems also influenced by environmental factors (up to 12% variance), including experiences unshared by twins [42] and neighborhood characteristics [43]. Admittedly, small relative-age effects on body mass and rate of maturation seem speculative, but have been reported [41].

### International comparison

There is an extensive literature about relative-age effects in multiple countries [5], but most samples were derived from the United States of America (USA) or United Kingdom (UK) [1,15]. One of the challenges in isolating relative-age effects is that their manifestation is contingent on mechanisms to group children in classes, which differ over time and place [44,45]. For example, the Dutch cohort under study was allocated over classes based upon an annual birthdate cutoff (pre/post October), and all children in each grade attended all courses together. In other systems children attend courses (*e*.*g*., language, math, or sports) subdivided on base of ability, a process called ‘setting’ or ‘banding’ [46], which may alleviate relative-age effects [21].

The influence of such apparently innocuous institutional differences becomes manifest in international comparisons. The 2006 Program for International Student Assessment (PISA) showed that up to 50% of the Dutch, Belgian, Austrian, or Czech 15-year olds were in a different grade than expected in a normative trajectory, compared to 12% in the USA, about 1% in the UK, and none in Japan or Finland [44]. This suggests that relative-age effects may manifest themselves via grade progression, that is, the possibility to allocate children to a higher or lower grade than the normative one, based on their abilities [27,44]. We expect that especially the less qualified relatively young are retained, whereas the highly qualified relatively old are accelerated, which we call ability streaming.

### The present study

A demonstration of persistent relative-age effects in adolescence, bestowed upon children by an adult-imposed structuring of their worlds, could lead to renewed awareness and prevention strategies among teachers, parents, and psychiatrists. We therefore aim to quantify associations between relative-age position effects and multiple outcome domains in early and middle adolescence in a Dutch sample. Relative-age effects were defined as the effects of intra-cohort age position adjusted for the actual age at the time of measurement (biological age). Only after adjustment for the additional developmental time granted to the relatively old at the time of measurements (a methodological artifact), we could observe the alleged *effects* of being months ahead (or behind), resulting from accumulating benefits and disadvantages *driven* by adolescents’ relative-age position.

We hypothesized unfavorable outcomes, in multiple domains, for the relatively young compared to the relatively old adolescents. More specifically, we expected to replicate relative age effects on school progress (H1), and tested whether relative-age predicted whether adolescents had repeated or skipped a grade (or went to special education). For the adolescents with a normative progress and the group who repeated a grade we tested whether relative-age predicted weight (H2a), pubertal status (H2b), school performance (H3), sport competence (H4), and peer status in terms of peer rejection (H5a) and popularity (H5b). These dependent variables enabled us to replicate earlier work on physical, intellectual, and social development in the Dutch culture (and educational context). In addition, we tested whether relative-age effects predicted mood (H6a, depressive symptoms) and temperamental fear and frustration (negative emotionality, H7a). Since persistent relative age effects imply a different developmental trajectory, and suggest accumulating change (*cf*. corresponsive principle), we also tested for change in depressive symptoms (H6b) and fear and frustration (H7b) between age 11 and 16. Because earlier work suggested associations between maternal socioeconomic status (SES) and month of birth [47–49], we ran all analyses without and with adjustment for family SES.

## Method

### Study design and sample

Data were collected as part of TRAILS, a large ongoing prospective cohort of Dutch adolescent followed to study the psychological, social and physical development of children towards adulthood [50]. The core aim was to unravel developmental pathways to psychological (ill) health. The study was approved by the Dutch Central Committee on Research Involving Human Subjects. The first measurement wave (T_1_) started in 2001, whereas data collection for the remaining waves (T_2_ to T_3_) took place at intervals of approximately 2.5 years. Written informed consent was collected from the parents at T_1_, whereas for T_2_ and T_3_ written informed consent was obtained from both parents and adolescents. The TRAILS design, sample selection, and data collection are described extensively elsewhere [50–52]. Briefly, participants born between October 1 in 1989 and September 30 in 1991 were selected from five municipalities in the north of the Netherlands. From a total of 2935 children, 2230 agreed to take part at T_1_ (response rate 76%, mean age 11.1, SD = 0.6, 51% girls). The response rates were 96% at T_2_ (N = 2149, mean age 13.6, SD = 0.5, 51% girls) and 81% at T_3_ (N = 1816, mean age 16.3, SD = 0.7, 52% girls). Non-response associated slightly with low socioeconomic background, male gender, low IQ and school performance, non-western ethnicity, and externalizing problems, but not with other emotional and behavioral problems [52]. At T_1_, the parents or guardians were interviewed at their homes, and handed in a previously sent questionnaire at that occasion. At T_2_ and T_3_, the questionnaire was sent to the parents or guardians by mail. At all three waves, the adolescents and their teachers completed the questionnaires at school.

### Measures

#### Relative-age

As outlined, when the TRAILS children entered school, children born between the first of October and the 30^th^ of September of the next year were allocated in the same age group in the Netherlands. Consequently, in a normative situation, children born in September were the youngest in a given grade (month 1), while children born in October were the oldest (month 12). This relative-age measure (1–12) was used as a continuous measure in our analysis.

#### School Progress

Information on school progress was collected from the schools before sample selection. Four categories were distinguished: (1) normal progression, (2) children who repeated a grade, (3) children who skipped a grade, and (4) children in special education. The groups of adolescents who had repeated one or two grades were merged because only nine adolescents had repeated twice.

#### Physical development

At *T*_2_, the length in centimeters (cm) and the weight in kilograms (kg, without shoes and heavy clothing) were measured. Body mass index (BMI) was calculated by dividing the weight (kg) by the square of the height (m^2^).

#### Pubertal status

At *T*_2_, pubertal status was measured with the Pubertal Development Scale (PDS, see [53]). The PDS assesses development on five (Tanner) characteristics, including growth spurt in height, skin changes, body hair in both boys and girls, breast development and menarche in girls, and voice change and facial hair growth in boys [54]. Each item was rated on a four-point scale (0 = not yet started, 1 = just started, 2 = going on for a while, 3 = passed that). In our analyses we used the mean of the five item scores. The PDS showed to be reliable with a Cronbach’s alpha of .75 for boys and .72 for girls.

#### School performance

At *T*_2_, teachers provided ratings for each child on history, geography, math, and natural sciences. The adolescent’s performance at school was operationalized as the composite of these marks rated on a five point scale, ranging from 1 = inadequate to 5 = outstanding. Cronbach’s alpha was .86.

#### Sport competence

In the Netherlands all children receive physical education. At *T*_2_, teachers were asked to rate the sport competence of each adolescent on a five-point scale (1 = inadequate, 2 = hardly adequate, 3 = adequate, 4 = good, and 5 = outstanding).

#### Peer status

At *T*_2_, social peer status (being popular or rejected) was assessed using a sociometric nomination procedure in classrooms with at least three TRAILS respondents (see [55]). Adolescents could nominate an unlimited number of classmates on a total of 18 questions, covering a wide range of issues and behaviors. For the purpose of this study, we selected the questions “which classmates do you like” and “which classmates do you totally dislike?”, and labeled the adolescents who scored above the 80^th^ percentile (i.e. got many nominations) as either popular or rejected [55]. Subsequently, we dummy coded adolescents who were regarded popular (= 1) against the adolescents without this specific peer nomination (= 0), and repeated this for rejected adolescents, to derive two sociometric status variables for 1007 adolescents (45.2% of the total sample; see Table 1). In total 149 adolescents were rated as popular (14.8%) and 155 as rejected (15.4%).

**Table 1:** Descriptive Statistics of the TRAILS Variables.

Variable	Wave	*N*	Range	Mean	*SD*	*Skewness*		*Kurtosis*	
						*z*	*SE*	*z*	*SE*
Relative Age		2230	1 to 12	6.20	3.44	0.11	0.05	-1.19	0.10
Relative Age (alternative)		794	1 to 12	8.02	2.99	-0.64	0.09	-0.37	0.17
Age	1	2230	10.01 to 12.58	11.11	0.56	0.49	0.05	-0.46	0.10
Fear	1	1982	1 to 5	2.42	0.73	0.33	0.06	-0.14	0.11
Frustration	1	1983	1 to 4.80	2.79	0.66	0.07	0.06	0.00	0.11
Depressive Symptoms	1	2024	0 to 2.31	0.48	0.36	1.07	0.05	1.33	0.11
SES	1	2188	-1.94 to 1.73	-0.05	0.80	-0.05	0.05	-0.80	0.11
Age in years	2	2149	12.15 to 15.15	13.57	0.53	0.00	0.05	-0.41	0.11
Length (cm)	2	2041	131 to 195	164.85	8.24	0.05	0.05	0.40	0.11
Weight (kg)	2	2030	29 to 134	52.84	11.08	1.23	0.05	3.81	0.11
BMI	2	2028	12.23 to 40.20	19.00	3.21	1.61	0.05	5.01	0.11
Physical Development	2	2087	1 to 20	9.34	3.38	-0.16	0.05	-0.59	0.11
Intellectual Development	2	1534	1 to 20	11.64	3.71	-0.46	0.06	-0.19	0.13
Social Status	2	1007	1 to 5	3.40	1.31	-0.79	0.08	-0.73	0.15
Sport Competence	2	1455	1 to 5	3.48	0.64	-0.33	0.06	0.99	0.13
Age	3	1819	14.69 to 18.69	16.28	0.71	0.73	0.06	-0.04	0.12
∆ Fear	1 to 3	1396	-3.57 to 4.12	0.05	1.04	0.18	0.07	0.65	0.13
∆ Frustration	1 to 3	1397	-4.07 to 3.11	0.02	0.99	-0.10	0.07	0.65	0.13
∆ Depressive Symptoms	1 to 3	1343	-3.63 to 4.94	0.00	1.06	0.29	0.07	1.44	0.13

*Note*. *N* = 2230 (50.8% women). ∆ = change score between *T*_1_ and *T*_3_; BMI = Body-Mass Index; cm = centimeter; *k* = number of categories; kg = kilogram; *N* = number of participants; *SD* = Standard Deviation; *SE* = Standard Error; SES = Socio-Economic Status; *T*_1_ = baseline wave; wave = measurement wave; z = z-scored or standardized (mean = 0, SD = 1), which means that z>1.64 is significant at *p* = .05, z> 2.33 at *p* = .01, and from z>3.10 at *p* = .001.

#### Depressive symptoms

Depressive symptoms were assessed at *T*_1_ and *T*_3_ with the Affective Problem scales of the Youth Self Report (YSR [56]) and parent-reported Child Behavior Checklist (CBCL), which cover depressive symptoms according to DSM-IV criteria with 13 items, including information on sadness, loss of pleasure, crying, self-harm, suicidal ideation, feelings of worthlessness, guilt, loss of energy, overtiredness, eating problems and sleeping problems [57–59]. Both scales are rated on a three-point scale ranging from 0 = never or not at all true to 2 = very often or very true. Cronbach’s alpha for the YSR was .72 at *T*_1_ and .78 at *T*_3_ and for the CBCL .68 at *T*_*1*_ and .76 at *T*_*3*_. In the analyses we used the combined mean scores of the CBCL and YSR scales.

#### Temperamental Fear and Frustration

Temperamental negative affectivity was assessed at *T*_1_ and *T*_3_with the Dutch parent version [60] of the revised Early Adolescent Temperament Questionnaire (EATQ-R [61]), which is based on the temperamental model by Rothbart et al. [62,63]. Earlier work in TRAILS showed the EATQ factor structure of the parent version to be superior to the child version [64]. Fear and frustration were measured with five questions rated on a five-point scale (ranging from 1 = almost never to 5 = almost always true). Cronbach’s alpha was .63 (*T*_1_) and .66 (*T*_3_) for Fear and .74 (*T*_1_) and .75 (*T*_3_) for Frustration.

#### Socioeconomic status

Socioeconomic status (SES) of the family of origin was the composite of five z-scored continuous variables measured at *T*_1_, including professional occupation and educational attainment of both parents/guardians, and household income.

### Plan of Analyses

Data cleaning, calculation of descriptives, and all analyses were performed in SPSS (version 20, SPSS Inc), Chicago, Illinois. To test whether relatively young or relatively old adolescents were more likely to repeat or skip a grade (H1), we performed bootstrapped multinomial logistic regression analyses with school progress as outcome (1 = normal progression, 2 = repeated grade, 3 = skipped grade, and 4 = special education). Normal school progress was used as reference category, and relative age effects on school progress were expressed in odds ratios.

Hypotheses H2 to H7 were tested with partial Pearson’s correlations (*r*_p_) adjusted for age at testing. In addition, we performed a series of linear regression analyses (with ordinary least squares estimators) in which relative-age predicted, respectively, length and weight (H2a), pubertal status (H2b), school performance (H3), sport competence (H4), depressive symptoms (H6a), fear and frustration (H7a), and change in depressive symptoms (H6b) or temperament (H7b), adjusted for age at testing. To obtain change scores for depressive symptoms and temperamental fear and frustration we subtracted *T*_1_ from *T*_3_ scores_._ To test relative-age effects on being rejected (H5a) and popular (H5b), we applied binary logistic regression analyses adjusted for age at testing.

All hypotheses were tested in adolescents with a normal school progress and in those who had repeated a grade, with the exception of H5 because of insufficient peer nomination data. We lacked power to test effects in the group that skipped a grade (S6 Table). Weight and BMI were non-normally distributed (see Table 1). The linear regression technique is known to remain valid in a sample of our size, even when dependent variables violate the “normality assumption” (see [65]). However, to ensure the robustness of our results, we bootstrapped all linear regression analyses (*k* = 10,000 with bias corrected confidence intervals, see [66]). We calculated the power for our regression analyses (S7 Table), and to enable comparison with other literature, we converted some results to Cohen’s *d* (standardized effect sizes), based on formulas derived from Borenstein [67] and Peterson [68]. To reduce family-wise alpha inflation, we only interpreted correlations that were significant at *p<* .01.

Finally, we performed two robustness checks. First, we repeated all analyses adjusted for family SES. Second, to circumvent the strong positive association between relative-age and biological age (which might obfuscate the meaning of our results), we composed an alternative relative age variable in which we combined the relative old children from grade 7 (month 7–12) and relative young from grade 8 (month 1–6). With this approach we derived a sample of 794 children with a normative school progress in whom the alternative relative-age variable still spanned 12 months, but was inversely associated with biological age (see Table 2). In this sample we conducted the same analyses (H2-7). Similar findings in both analyses would strongly suggest that the results are not an artifact of the association between relative age and biological age.

**Table 2:** Pearson correlations among all study variables.

	Wave		1.	2.	3.	4.	5.	6.	7.	8.	9.	10.	11.	12.	13.	14.	15.	16.	.17
1.	Relative Age		-																
2.	Length	2	**.15** ^***^	-															
3.	Weight	2	**.13** ^***^	**.62** ^***^	-														
4.	BMI	2	**.08** ^***^	**.19** ^***^	**.88** ^***^	-													
5.	Physical Dev.	2	**.15** ^***^	**.46** ^***^	**.43** ^***^	**.29** ^***^	-												
6.	Intellectual Dev.	2	-.01	.02	-.01	-.03	.03	-											
7.	Popular status	2	.00	-.04	-.08	-.08	.08	.10^*^	-										
8.	Rejected status	2	.06	.04	.08	.10	.06	.02	-	-									
9.	Sport Competence	2	-.02	-.05	**-.16** ^***^	**-.17** ^***^	-.02	**.28** ^***^	.12^*^	-.06	-								
10.	Fear	1	-.02	-.03	.03	.06^*^	.03	-.04	.10^*^	.02	-.03	-							
11.	Frustration	1	.01	.04	**.07** ^**^	**.07** ^**^	.02	-.05	-.06	**.11** ^**^	-.05	**.31** ^***^	-						
12.	Depressive Sx	1	-.01	-.03	.03	.04	.01	-.02	-.10^*^	**.17** ^***^	**-.12** ^***^	**.29** ^***^	**.32** ^***^	-					
13.	ΔFear	1–3	-.01	-.02	-.02	.01	.03	-.06^*^	-.02	-.02	-.03	**-.51** ^***^	**-.08** ^**^	-.07^*^	-				
14.	ΔFrustration	1–3	.03	-.02	-.02	-.02	.02	-.07^*^	.04	-.06	-.05	**-.11** ^***^	**-.49** ^***^	**-.08** ^**^	**.26** ^***^	**-**			
15.	Δ Depressive Sx	1–3	.03	.00	.05	**.07** ^**^	.**11** ^***^	.00	.06	-.08	-.04	-.08^**^	**-.08** ^**^	**-.52** ^***^	**.19** ^***^	**.18** ^***^			
16.	Rel. Age (control)		**1.00** ^***^	.02	.07	.08^*^	.02	.01	.03	**-.07** ^***^	.01	-.01	.05	.03	-.04	.04	.01		
17.	Biological Age		**.53** ^***^	**.15** ^***^	**.14** ^***^	**.09** ^***^	**.16** ^***^	-.04	-.02	**-.02**	-.03	**-.07** ^**^	-.04	-.05^*^	.08^**^	.06^*^	.07^*^	**-.28** ^***^	
18.	SES	1	-.03	.05^*^	**-.08** ^***^	**-.13** ^***^	.00	**.22** ^***^	.01	.00	**.14** ^***^	**-.08** ^***^	**-.06** ^**^	**-.07** ^***^	**-.09** ^***^	-.03	-.02	**-.10** ^**^	**-.03**

*Note*. *N* = 2230 (50.8% women). Rel. Age = Relative Age; ∆ = change; SES = socioeconomic status of the family of origin. Table 1 gives details (*e*.*g*., ages). For popular and rejected social status we report biserial correlations (*e*.*g*. being popular or rejected or not), because the scale was artificially dichotomous. Partial correlations adjusted for real age at testing, which show the relative age effects, are presented in Table 4. Significance

^***^ *p*<.001

^**^ *p*<.01 (reported in bold)

^*^ *p*<.05, two-tailed.

## Results

### Descriptive Statistics

Descriptive statistics of all variables used in this study are reported in Table 1. The correlations shown in Table 2 indicate that relatively old adolescents were somewhat larger, heavier, and in a more advanced pubertal development stage than the relatively young adolescents.

### School progress

**Fig 1:**
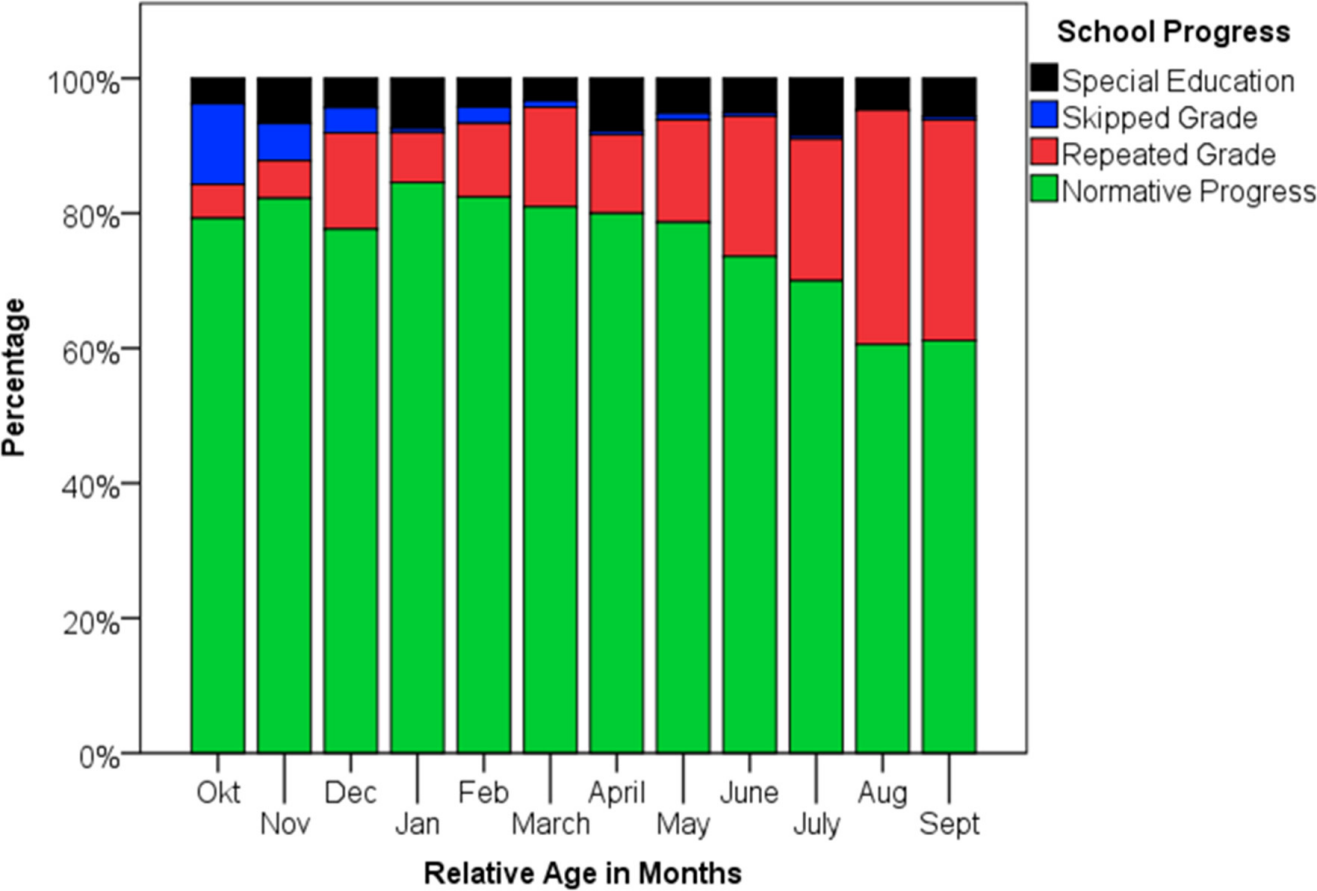
School Progress Stratified Over Relative Age Position.

Relative-age effects on school progress are reported in Table 3. For each additional month, relative-age was associated with 17% lower odds of grade repetition and a 47% increased odds of skipping a grade (see also Fig 1). The relatively young quartile (July to Sept) was almost four times more likely to repeat a grade (29.6% *vs*. 8.2%) and over twenty times less likely to skip a grade (0.3% *vs*. 7%) than the relatively old quartile (Oct to Dec); all details can be found in S1 Table. Compared to adolescents with a normative school progress, adolescents who repeated a grade were on average 8 weeks younger, while adolescents who skipped a grade were on average 14 weeks older (75% of the latter were relatively old). As shown in Table 3, no association was found for special education (*d≈* 0.03 [95% CI = -0.06 to 0.01]). School progress itself was related to SES (see S5 Table and S6 Table), and after adjustment for SES, the relative age effects on skipping a class became slightly stronger, and the effect on special education significant. This may reflect the role SES played in the decision who skipped a class and who went to special education (see S5 Table, S6 Table). However, since only 48 adolescents skipped a class and 124 went to special education, which are rather small samples, these results warrant replication and cautious interpretation.

**Table 3:** Relative age effects on school progress (normative development is reference).

		Adj. for real age^a^		Adj. for real age & SES^b^	
Binary Outcome:	N	OR	(95% CI)	OR	(95% CI)
Repeated grade	377	0.83^***^	(0.80 to 0.86)	0.82^***^	(0.79 to 0.86)
Skipped grade	48	1.47^***^	(1.30 to 1.67)	1.66^***^	(1.44 to 1.92)
Special education	124	0.96	(0.91 to 1.01)	0.81^***^	(0.75 to 0.87)

*Note*. *N* = 2230 (50.8% women).

^a^ The odds are based on bootstrapping (*k* = 10.000)

^**b**^ odds are not based on bootstrapping. CI = bias corrected confidence interval; OR = Ratio of the probability that an event will happen to all possible cases for that event. Adolescents who repeated a grade were almost three times more often from the lowest than the highest SES quartile (22.7% *vs*. 8.6%), but adolescents who skipped a grade were three times more often from the highest than the lowest SES quartile (3.1% *vs*. 0.9%), see S5 Table and S6 Table.

### Adolescents with a normative school progress

Recall that all the following results were adjusted for age at testing. Partial correlations and linear regression models in the subgroup of adolescents with a normative school progress (75.4%, *n* = 1681) in Table 4 showed that relative-age effects predicted temperamental frustration (*d≈* 0.22); but this effect disappeared after adjustment for SES (S2 Table). We further observed that relative-age predicted social rejection (OR = 1.08, *p*< .01), but was unrelated to popularity (OR = 0.96, see S3 Table). This rather small relative-age effect on rejection followed a u-shape, favouring the middle quartiles (17.5%, 11.0%, 12.4%, 17.9%, respectively, see S4 Table). All other associations were absent. Because relative-age correlated *r* = .53 with biological age (Table 2) we calculated an alternative relative-age sample to check our results for robustness (see method section). In this alternative sample relative age correlated *r* = -.28 with biological age (Table 2), but led to similar results (Table 4). In sum, the key observation was the absence of substantive relative-age effects in the adolescents with a normative school progress.

**Table 4:** Relative Age Effects, Adjusted for Actual Age, as Predictor of Multiple Domains, for Adolescents with a Normative School Progress (*n* = 1681) and Adolescents who had Repeated a Grade (*n* = 377).

	Normative school progress				Children who repeated a class			Alternative relative age sample composition		
Variable	Wave	*r*_*p*_	B	95%CI	*r*_*p*_	B	95% CI	*r*_*p*_	B	95% CI
Length (cm)	2	-.04	-0.10	-0.24 to 0.04	.03	0.09	-0.23 to 0.43	-.03	-0.08	-0.29 to 0.13
Weight (kg)	2	-.03	-0.10	-0.28 to 0.08	.09	0.57^*^	0.07 to 1.09	.01	0.05	-0.21 to 0.30
BMI	2	-.01	-0.01	-0.06 to 0.04	.11	0.19^*^	0.04 to 0.36	.00	0.04	-0.03 to 0.11
Pubertal status	2	-.01	-0.04	-0.10 to 0.02	.09	0.07	-0.07 to 0.19	-.02	-0.03	-0.11 to 0.05
Intellectual Development	2	.04	0.02	-0.00 to 0.04	-.14^*^	-0.05^*^	-0.10 to -0.01	.05	0.04	-0.07 to 0.15
Sport Competence	2	.01	0.00	-0.02 to 0.02	-.02	-0.01	-0.05 to 0.04	.02	0.00	-0.00 to 0.00
Fear	1	-.02	0.01	-0.01 to 0.02	.10	0.04	0.00 to 0.08	-.03	**-0.01** ^***^	-0.01 to -0.01
Frustration	1	.05^*^	0.01^*^	0.01 to 0.02	.05	0.02	-0.03 to 0.06	.03	0.01	-0.01 to 0.02
Depressive symptoms	1	.02	0.00	-0.01 to 0.01	**.18** ^***^	**0.07** ^**^	0.02 to 0.11	.03	0.00	-0.01 to 0.01
∆ Fear	1–3	-.05	-0.02	-0.04 to 0.00	-.11	-0.06	-0.13 to 0.02	-.06	**-0.02** ^***^	-0.02 to -0.02
∆ Frustration	1–3	-.01	-0.00	-0.23 to 0.02	-.04	-0.03	-0.09 to 0.04	.02	0.01^*^	0.01 to 0.01
∆ Depressive symptoms	1–3	.01	0.00	-0.02 to 0.02	-.08	-0.03	-0.09 to 0.00	-.01	**-0.01** ^***^	-0.01 to -0.01

The alternative relative age sample composition comprised relative old children from grade 7 and relative young from grade 8 with a normative school progress (*n* = 794). *Note*. ∆ = change between *T*_1_ (age 11) and *T*_3_ (Age 16); BMI = body mass index; *r*_p_ = partial correlations between relative age and outcome, adjusted for real age at time of testing. Regression estimates were bootstrapped (*k* = 10,000 with bias corrected intervals), and indicate change in outcome per month in relative age, after adjustment for age at testing. Note that for change variables we also adjusted for change in age between *T*_1_ and *T*_3_. Details on all measures and procedures can be found in the method section. All correlations between all variables are given in Table 2, and SES-adjusted regression estimates in the supplementary (S2 Table). Significance

^***^ *p*<.001

^**^ *p*<.01 (in bold)

^*^ *p*<.05, two-tailed.

### Adolescents who had repeated a grade

Though adolescents who had repeated a grade (16.9%, *n* = 377) were inherently relatively old compared to their new peers (with a normative school progress), relative-age effects might still play a role for adolescents who repeated a grade. Partial correlations, presented in Table 4, showed slightly lower intellectual ability and more depressive symptoms for the relatively old adolescents who repeated a grade; a reversed relative-age effect. As shown in Table 4, linear regression models showed that relative older adolescents were heavier (*d≈* 0.39), had a higher BMI (*d≈* 0.42), lower intellectual ability (*d≈* 0.35), and reported more depressive symptoms (*d≈* 0.50). These results persisted after adjustment for SES (S2 Table).

### Post-hoc test of moderation by gender

Our results failed to support relative age effects in adolescents with a normative school progress, while these effects are commonly reported in the literature. One explanation may be that relative-age effects are stronger in males [15]. To test whether relative age effects were moderated by gender we fit all models for adolescents with a normative school progress (a) adjusted for gender and (b) including the interaction term (relative age * gender), see S7 Table. No gender effects were observed.

## Discussion

In this study we tested effects of intra-classroom relative age position on multiple domains of functioning and well-being in adolescence. Three key observations merit further discussion. First, we observed substantial relative-age effects on school progress; relatively young adolescents repeated a grade about four times more often than the relatively old, who in turn were over 20 times more likely to skip a grade. These observations align with our first hypothesis (H1), and replicate earlier studies [1,12,27].

Second, in adolescents with a normative school progress (75.4%), no substantive relative-age effects were observed. Although we observed a small effect on peer rejection (H5a), which might be a chance finding, we could not replicate previously reported relative-age effects on weight (H2a), pubertal status (H2b), school performance (H3), sport-competence (H4), or peer status in terms of popularity (H5b). Neither did we observe substantial effects on depressive symptoms (H6a), temperamental negative affectivity (H7a), and changes in depressive symptoms (H6b) or negative affectivity (H7b) between age 11 and 16. Third, in the subgroup of adolescents who had repeated a grade (16.9%), inverse relative-age effects were observed; the relatively young were thinner (weight and BMI), had higher school marks, and reported less depressive symptoms than their relatively older peers.

Administrators seem more willing to retain the relatively young and consider this factor in retention decisions (*cf*. S5 Table and S6 Table), which could explain the inverse-relative age effects in children who repeated a grade.

### Relative-age effects

The absence of substantial relative-age effects in adolescents with a normative school progress is surprising, given that the existing literature reports substantial effects [1,3,27,34], which also covers adolescents between age 11 and 17 [13,15,69]. Some studies reported that relative-age effects reverse for relatively young adolescents who managed to stay in their initial cohort [44,70], called the extended Akerlof/Kranton model [13]. We also did not replicate this reverse relative-age effect.

Another common argument is that relative-age advantages erode when full maturity in the specific system has been reached, in contrast to advantages conferred by genes [13,24]. Some studies indeed reported this dissipation of differences [1,71]. However, many other studies reported relative age effects on health, educational attainment, earnings, and mortality that persisted into adulthood [5,12,26]. This suggests that relative age advantages modulate personal and social development such that perpetuating mechanisms “get under the skin”, for example, via identity-formation [16,17], opportunity costs [4,5,24,26], cognitive networks [39], and/or development of (soft) skills [14,15]. We may interpret the substantial relative age effects on school progress along similar lines, an amplification of small differences.

### Ability streaming

The absence of substantive relative-age effects in adolescents with a normative school progress may be a Dutch cultural artifact. In our sample of adolescents at age 11 about 20% had repeated or skipped a grade (another 6% went to special education), which is already more than the 1% in the UK and 12% in the USA at age 15 [44]. Furthermore, typical American school classes include children with a range of learning needs or abilities (*e*.*g*., lower level for language than math), while Dutch children are sorted over general ability groups. There has been argued that these latter systems (such as the Dutch) intensify relative age effects [21]. Our results, however, suggest that ability streaming might have diluted the relative-age effects in the adolescents with a normative school progress. It may be that positive spillover from relatively older peers thereafter result in a net zero effect for the relatively young [45]. Alternative (and convergent) explanations are that ii) the Dutch environment is less competitive (which is a known moderator of relative age effects [15]), iii) that combined classrooms with multiple grades dilute effects, or iv) that relative age effects do not exist.

Our ability streaming hypothesis aligns in a slightly unexpected way with the hypothesized reciprocal feedback loops in which individuals shape (select/evoke) their environments to their propensities, in analogy to the corresponsive principle [22,23] and Dickens-Flynn model [24,25]. The corresponsive principle postulates that intra-classroom relative-age position effects can amplify small intrinsic differences, a process that may be catalyzed by strong ability streaming (via its outcome, that is, a change of grade), which results in a qualitative different classroom environment.

The ability streaming hypothesis may also explain the reversed relative-age effects we observed in adolescents who repeated a grade; with increasing relative-age low innate potential (or individual maturity) rather than chronological age (the cultural relative-age artifact) becomes the preeminent reason for grade retention, which is reflected in lower school performance and more depressive symptoms for the relative old than the relative young. Additionally, relatively young retenders also lost their undesirable intra-cohort position (they became relatively old), whereas the relatively old became *conspicuously* old [27]. Negative long-term effects of grade retention have been reported before [72].

### Strengths and limitations

Our results should be interpreted in light of the following strengths and limitations. A notable strength of this study is the large sample of adolescents from the general Dutch population. We are quite confident that substantial relative age effects are absent in our sample, because our power calculations (S7 Table) for our specific regression analyses indicated that we would observe effects that explained 1% of the variance in each outcome variable in adolescents with normative school progress (≥*d* = 0.18) and 3% in the group who repeated a grade (≥*d* = 0.34). Moreover, we repeated analyses with our alternative relative-age variable. Although relative age was composed differently, and showed a negative association with biological age (*r* = -.28 versus *r* = .53), the results were similar.

A limitation is that we did not cluster for region, school, and classroom, and we could not adjust for intelligence (but we used school grades). Furthermore, our adjusted regression analyses lack the rigor of an experimental design. The biggest limitation, however, is our lack of knowledge about *why* children repeated a grade. For example, some studies reported that in other counties some high SES parents deliberately delay their relatively young child’s entry into school (“academic redshirting”) to create a favorable position as a relatively old child [5,15,73]. This suggests cumulative disadvantages for relatively young children from a low SES background [14]. However, note that we did not observe more relatively young adolescents from families in the low SES quartile (S1 Table), which would suggest a strategic delay in affluent strata, and our results remained unchanged after adjustment for family SES (except the effect on frustration). Furthermore, grade repetition was based upon school report. Notably, alternative explanations like season-of-birth effects have been refuted in earlier work, because relative age effects were observed in both hemispheres [5,15] and remained after statistical adjustment for seasonal effects [11]. The suggestion that relative age effects were moderated by gender (i.e. stronger for males [15]) was not supported by our post-hoc test.

Nowadays Dutch elementary schools often rank (and instruct) children before age 7 on base of ability (from the most able “sun” children, via promising “rockets” and average “moons” to the least bright “star” children), and theorists have argued that such processes alleviate relative-age effects [21,46]. This may be true in terms of school progress, but the effects of explicit (ability) ranking on children’s mental development, and its interaction with relative age, remain to be verified [7,74].

### Conclusion

We studied the relative-age effect bestowed upon children by an adult-imposed structuring of their worlds, a cultural artifact that may modulate the development of their innate abilities. Our preeminent observation is that intra-classroom position influenced school progress, and rendered relatively young adolescents more likely to repeat a grade. Second, in adolescents with a normal school progress we could not replicate substantial relative-age effects (i.e., developmental differences due to age position) in terms of physical and psychosocial development and well-being. We argued that this absence of relative-age effects in adolescents with a normative school progress might reflect a “wash-out effect” due to ability streaming (i.e., nonrandom sorting of oldest and youngest). This ability streaming (mainly grade retention; 20% of the sample) may distinguish the Dutch sample from earlier studies in Britain and the USA. Third, in the subgroup of adolescents who repeated a grade, reverse relative-age effects were observed; the relatively old adolescents performed worse in school and reported more depressive symptoms than their relatively younger peers did. This may reflect a relative age effect on the decision threshold for grade retention (amplified by low SES). Such effects might motivate policy makers to make Dutch education more flexible, *e*.*g*. by enabling children to study difficult courses on their ability levels, rather than repeating a whole year curriculum. Future studies might explore the mechanisms by which relative-age effects may get internalized in more detail, and cerebrate models that explain alleged cultural differences in their effects, such as their absence in the Dutch educational system.

## Supporting Information

S1 Table Frequencies for school progress and socioeconomic status stratified over relative age. (DOCX)

S2 Table Relative age effects on multiple domains. (DOCX)

S3 Table Relative age as the predictor of social status. (DOCX)

S4 Table Social status stratified over relative age position and school progress. (DOCX)

S5 Table School progress stratified over four quartiles of socioeconomic status. (DOCX)

S6 Table Socioeconomic status stratified over school progress. (DOCX)

S7 Table Power calculations. (DOCX)

S8 Table Interaction by gender. (DOCX)

S9 Table Percentage of children with a specific relative age position by socioeconomic status. (DOCX)

S1 Data Relative Age Effect. (SAV)
