# Supplementary material for: Relative Age Effects in Dutch Adolescents: Concurrent and Prospective Analyses
Source: PLoS One. 2015 Jun 15;10(6):e0128856. doi: 10.1371/journal.pone.0128856 (PMC4468064; doi:10.1371/journal.pone.0128856)
Supplement: S1 Table — (DOCX) [file pone.0128856.s001.docx]

**S1 Table.**

Frequencies for school progress and socioeconomic status stratified over relative age

|  |  |  |  | **Relative Young** | | **2^nd^ Quartile** | | **3^d^ Quartile** | | **Relative Old** | |
| --- | --- | --- | --- | --- | --- | --- | --- | --- | --- | --- | --- |
| Relative age: |  |  |  | 1 to 3 | | 4 to 6 | | 7 to 9 | | 10 to 12 | |
| Birth month: |  |  |  | Sept, Aug, July | | June, May, April | | March, Feb, Jan | | Dec, Nov, Oct | |
| Total sample |  | 2230 | 100.0% | 624 | 100% | 569 | 100% | 537 | 100% | 500 | 100% |
| Normative development |  | 1681 | 75.4% | 398 | 63.8% | 441 | 77.5% | 443 | 82.5% | 399 | 79.8% |
| Repeated grade |  | 377 | 16.9% | 185 | 29.6% | 90 | 15.8% | 61 | 11.4% | 41 | 8.2% |
| Skipped grade |  | 48 | 2.2% | 2 | 0.3% | 4 | 0.7% | 7 | 1.3% | 35 | 7.0% |
| Special education |  | 124 | 5.5% | 39 | 6.3% | 34 | 6.0% | 26 | 4.8% | 25 | 5.0% |
| Low SES quartile |  | 547 | 25.0% | 135 | 24.7% | 142 | 26.0% | 149 | 27.2% | 121 | 22.1% |
| High SES quartile |  | 547 | 25.0% | 150 | 27.4% | 141 | 25.8% | 139 | 25.4% | 117 | 21.4% |

*Note. N*= 2230 (50.8% women); %= percentage; SES= socioeconomic status; Relative age in quartiles.
