# Supplementary material for: Relative Age Effects in Dutch Adolescents: Concurrent and Prospective Analyses
Source: PLoS One. 2015 Jun 15;10(6):e0128856. doi: 10.1371/journal.pone.0128856 (PMC4468064; doi:10.1371/journal.pone.0128856)
Supplement: S2 Table — (DOCX) [file pone.0128856.s002.docx]

**S2 Table**

Relative age effects on multiple domains

|  |  | **Normal School Progress** | | | **Repeated Grade** | | |
| --- | --- | --- | --- | --- | --- | --- | --- |
|  |  | Relative age effect | | | Relative age effect | | |
| Variable | Wave | *r_p_* | B | 95% CI | *r_p_* | B | 95% CI |
| Length (cm) | 2 | -.04 | -0.10 | (-0.24 to 0.04) | -.01 | 0.09 | (-0.25 to 0.42) |
| Weight (kg) | 2 | -.04 | -0.13 | (-0.30 to 0.05) | .08 | 0.60^*^ | (0.02 to 1.11) |
| BMI | 2 | -.02 | -0.02 | (-0.07 to 0.03) | .10 | 0.19^*^ | (0.03 to 0.36) |
| Pubertal status | 2 | -.02 | -0.04 | (-0.10 to 0.02) | .08 | 0.07 | (-0.07 to 0.19) |
| Intellectual Development | 2 | .04 | 0.06 | (-0.01 to 0.13) | -.12 | -0.19^*^ | (-0.36 to -0.02) |
| Sport Competence | 2 | .01 | 0.00 | (-0.01 to 0.02) | -.01 | -0.00 | (-0.03 to 0.03) |
| Fear | 1 | .01 | 0.01 | (-0.01 to 0.02) | .09 | 0.04 | (-0.00 to 0.08) |
| Frustration | 1 | .05 | 0.02 | (-0.00 to 0.03) | .04 | 0.02 | (-0.03 to 0.06) |
| Depressive symptoms | 1 | .01 | 0.00 | (-0.02 to 0.02) | **.18^***^** | **0.02^**^** | (0.02 to 0.11) |
| ∆ Fear | 1-3 | -.06 | -0.02 | (-0.04 to 0.00) | -.11 | -0.06 | (-0.13 to 0.02) |
| ∆ Frustration | 1-3 | -.01 | -0.00 | (-0.03 to 0.02) | -.04 | -0.03 | (-0.09 to 0.04) |
| ∆ Depressive symptoms | 1-3 | .01 | 0.00 | (-0.02 to 0.02) | -.08 | -0.03 | (-0.09 to 0.04) |

*Note*. Relative age effects have been adjusted for actual age at testing and socioeconomic status (SES) of the family of origin, for adolescents with a normative school progress (*n*=1681) and adolescents who repeated a grade (*n*= 377); ∆= change between *T*_1_ (age 11) and *T*_3_ (Age 16); BMI= body mass index; *r*_p_= partial correlations between relative age and outcome, adjusted for real age at time of testing. Regression estimates were bootstrapped (*k*=10,000 with bias corrected intervals), and indicate change in outcome per month in relative age, after adjustment for age at testing. Note that for change variables we also adjusted for change in age between *T*_1_ and *T*_3_. Details on all measures and procedures can be found in the method section. All correlations between all variables are given in Table 2.

Significance: ^***^*p*<.001, ^**^*p*<.01 (in bold), ^*^*p*<.05, two-tailed.
