# Supplementary material for: Relative Age Effects in Dutch Adolescents: Concurrent and Prospective Analyses
Source: PLoS One. 2015 Jun 15;10(6):e0128856. doi: 10.1371/journal.pone.0128856 (PMC4468064; doi:10.1371/journal.pone.0128856)
Supplement: S3 Table — (DOCX) [file pone.0128856.s003.docx]

**S3 Table**

Relative age as the predictor of social status

| **School progress** | **Social Status** | ***r*_p_** | **OR** | **(95% CI)** | ***p*** | **B** | **(95% CI)** |
| --- | --- | --- | --- | --- | --- | --- | --- |
| Normative | Popular | -.05 | 0.96 | (0.90 to 1.02) | .18 | -0.46 | (-0.11 to 0.02) |
|  | Rejected | **.08^**^** | 1.08 | (1.02 to 1.16) | .02 | 0.08 | (0.01 to 0.15) |
| Repeated a grade | Popular | .14 | 1.14 | (0.84 to 1.53) | .42 | 0.13 | (-0.83 to 0.60) |
|  | Rejected | .09 | 1.08 | (0.86 to 1.35) | .53 | 0.08 | (-0.19 to 0.36) |

*Note.* *N*=907 (53.1% women). The table shows the odds of being popular or rejected (normative development is reference) in adolescents with a normative school progress and adolescents who repeated a grade, adjusted for real age at testing. In total 137 (15.1%) adolescents were rated as popular and 132 (14.6%) as rejected. Regression estimates are based on bootstrapping (*k*= 10,000), the confidence intervals (95% CI) bias corrected; OR= Ratio of the probability that an event will happen to all possible cases for that event; *r*_p_= partial correlations between relative age and outcome, adjusted for real age at time of testing. The values for the presented correlations and regression remained almost identical after additional adjustment for family SES. Note that the estimations for the group who repeated a grade are based on very few subjects (see Table S6), therefore unreliable, thus not reported in the manuscript. Significance ^***^*p*< .001, ^**^*p*< .01 (in bold), ^*^*p*< .05, two-tailed.
