# Supplementary material for: Relative Age Effects in Dutch Adolescents: Concurrent and Prospective Analyses
Source: PLoS One. 2015 Jun 15;10(6):e0128856. doi: 10.1371/journal.pone.0128856 (PMC4468064; doi:10.1371/journal.pone.0128856)
Supplement: S4 Table — (DOCX) [file pone.0128856.s004.docx]

**S4 Table.**

Social status stratified over relative age position and school progress

|  | **Normative school progress** | | | | | **Repeated a grade** | | | | |
| --- | --- | --- | --- | --- | --- | --- | --- | --- | --- | --- |
|  | Total | Rejected | | Popular | | Total | Rejected | | Popular | |
|  |  | *n* | % | *n* | % |  | *n* | % | *n* | % |
| Relatively young | 211 | 37 | 17.5% | 30 | 14.2% | 25 | 5 | 20.0% | 3 | 12.0% |
| Second | 245 | 27 | 11.0% | 42 | 17.1% | 13 | 3 | 23.1% | 1 | 7.7% |
| Third | 233 | 29 | 12.4% | 37 | 15.9% | 12 | 3 | 25.0% | 1 | 8.3% |
| Relatively old | 218 | 39 | 17.9% | 28 | 12.8% | 8 | 2 | 25.0% | 1 | 12.5% |
| Total | 907 | 132 | 14.6% | 137 | 15.1% | 58 | 13 | 22.4% | 6 | 10.3% |

*Note.* Relative age was reported in quartiles. The frequencies of subjects with a rejected and popular status stratified for adolescents with a normative school progress and for the group of adolescents who repeated a grade.

*n*= number of subjects.
