# Supplementary material for: Relative Age Effects in Dutch Adolescents: Concurrent and Prospective Analyses
Source: PLoS One. 2015 Jun 15;10(6):e0128856. doi: 10.1371/journal.pone.0128856 (PMC4468064; doi:10.1371/journal.pone.0128856)
Supplement: S5 Table — (DOCX) [file pone.0128856.s005.docx]

**S5 Table.**

School progress stratified over four quartiles of socioeconomic status

|  | **Normative**  **development** | | **Repeated a**  **grade** | | **Skipped a**  **grade** | | **Special education** | |
| --- | --- | --- | --- | --- | --- | --- | --- | --- |
| **SES** | *n* | % | *n* | % | *n* | % | *n* | % |
| Low SES | 357 | 65.3% | 124 | 22.7% | 5 | 0.9% | 61 | 11.2% |
| Second Q | 394 | 72.0% | 117 | 21.4% | 4 | 0.7% | 32 | 5.9% |
| Third Q | 422 | 77.1% | 83 | 15.2% | 21 | 3.8% | 21 | 3.8% |
| High SES | 479 | 87.6% | 47 | 8.6% | 17 | 3.1% | 4 | 0.7% |
| Total: | 1652 |  | 371 |  | 47 |  | 118 |  |

*Note. n*= number of subjects; Q= Quartile. SES= Socioeconomic Status; The adolescents who repeated a grade were almost three times more often from the lowest than the highest SES quartile (22.7% *vs.* 8.6%), but adolescents who skipped a grade were three times more often from the highest than the lowest SES quartile (3.1% *vs.* 0.9%). For school progress in terms of months of relative age and SES, see S9 Table.
