# Supplementary material for: Relative Age Effects in Dutch Adolescents: Concurrent and Prospective Analyses
Source: PLoS One. 2015 Jun 15;10(6):e0128856. doi: 10.1371/journal.pone.0128856 (PMC4468064; doi:10.1371/journal.pone.0128856)
Supplement: S6 Table — (DOCX) [file pone.0128856.s006.docx]

**S6 Table.**

Socioeconomic status stratified over school progress

|  | **Low SES** | | **Second Q** | | **Third Q** | | **High SES** | | Total | |
| --- | --- | --- | --- | --- | --- | --- | --- | --- | --- | --- |
|  | *n* | % | *n* | % | *n* | % | *n* | % | *n* | % |
| Normative | 357 | 21.6% | 394 | 23.8% | 422 | 25.5% | 479 | 29.0% | 1652 | 100% |
| Repeated a grade | 124 | 33.4% | 117 | 31.5% | 83 | 22.4% | 47 | 12.7% | 371 | 100% |
| Skipped a grade | 5 | 10.6% | 4 | 8.5% | 21 | 44.7% | 17 | 36.2% | 47 | 100% |
| Special education | 61 | 51.7% | 32 | 27.1% | 21 | 17.8% | 4 | 3.4% | 118 | 100% |

*Note*. SES= Socioeconomic status in quartiles; In the adolescents who repeated a class, more adolescents from a high than low SES background were relative young (month 1-3: 61.7% *vs*. 46.8%, respectively), while few were relatively old (month 9-12: 2.1% *vs*. 10.4%, respectively, leaving a middle of 36.2% vs. 57.2%); n= number of participants.
