# Supplementary material for: Relative Age Effects in Dutch Adolescents: Concurrent and Prospective Analyses
Source: PLoS One. 2015 Jun 15;10(6):e0128856. doi: 10.1371/journal.pone.0128856 (PMC4468064; doi:10.1371/journal.pone.0128856)
Supplement: S7 Table — (DOCX) [file pone.0128856.s007.docx]

**S7 Table.**

Power calculations

| Category | Reasonably precise estimates beyond: | Given 80% power |
| --- | --- | --- |
| Normative school progress | Cohen’s *f*^2^= 0.01 | R^2^ is about (~) 0.01, *d*= ~0.18, 2 predictors, α= .05, and *N*≥ 1100 for all outcomes |
| Repeated a grade | Cohen’s *f*^2^= 0.03 | R^2^= ~0.03, *d*= ~0.34, given 80% power, 2 predictors, α= .05, *N*≥ 320 for all outcomes |
| Skipped a grade | Cohen’s *f*^2^= 0.24 | R^2^= 0.19, *d*= ~0.98, *N*= 48 |

*Note*. We calculated the power of all specific linear regression analyses. The results indicate that we lacked the power to test for relative age effects in the group that skipped a grade. These post-hoc power calculations indicate that we obtained reasonably precise estimations in our study, and we are therefore confident that substantial relative age effects are indeed absent.

Reference: Selya, A. S., Rose, J. S., Dierker, L. C., Hedeker, D., & Mermelstein, R. J. (2012). A practical guide to calculating Cohen’s f2, a measure of local effect size, from PROC MIXED. *Frontiers in Psychology, 3*(111) doi:10.3389/fpsyg.2012.00111
