# Supplementary material for: Relative Age Effects in Dutch Adolescents: Concurrent and Prospective Analyses
Source: PLoS One. 2015 Jun 15;10(6):e0128856. doi: 10.1371/journal.pone.0128856 (PMC4468064; doi:10.1371/journal.pone.0128856)
Supplement: S8 Table — (DOCX) [file pone.0128856.s008.docx]

**S8 Table.**

Interaction by gender

|  |  | **Relative age effect, adjusted**  **for biological age and gender** | | | | **Interaction: Relative age**  **effect * gender** | | |
| --- | --- | --- | --- | --- | --- | --- | --- | --- |
| Variable | Wave | Beta | B | 95% CI | Beta | | B | 95% CI B |
| Length (cm)† | 2 | -0.05 | -0.11 | (-0.26 to 0.03) | 0.16^**^ | | 0.31^**^ | (0.08 to 0.54) |
| Weight (kg) | 2 | -0.03 | -0.10 | (-0.27 to 0.08) | 0.10 | | 0.26 | (-0.04 to 0.57) |
| BMI | 2 | 0.00 | 0.00 | (-0.07 to 0.03) | -0.02 | | -0.02 | (-0.10 to 0.06) |
| Pubertal status | 2 | 0.02 | 0.01 | (-0.01 to 0.02) | 0.09 | | 0.02 | (-0.00 to 0.04) |
| Intellectual Development | 2 | 0.07 | 0.02^*^ | (0.00 to 0.04) | 0.04 | | 0.01 | (-0.02 to 0.05) |
| Sport Competence | 2 | 0.02 | 0.01 | (-0.01 to 0.02) | -0.10 | | -0.03 | (-0.06 to 0.01) |
| Fear | 1 | 0.03 | 0.01 | (-0.01 to 0.03) | 0.03 | | 0.01 | (-0.02 to 0.04) |
| Frustration | 1 | 0.06 | 0.02 | (-0.00 to 0.04) | 0.04 | | 0.01 | (-0.02 to 0.04) |
| Depressive symptoms | 1 | 0.02 | 0.01 | (-0.01 to 0.02) | 0.03 | | 0.01 | (-0.02 to 0.04) |
| ∆ Fear | 1-3 | -0.05 | -0.02 | (-0.04 to 0.01) | 0.04 | | 0.01 | (-0.03 to 0.05) |
| ∆ Frustration | 1-3 | -0.00 | 0.00 | (-0.02 to 0.02) | 0.09 | | 0.02 | (-0.01 to 0.06) |
| ∆ Depressive symptoms | 1-3 | 0.02 | 0.01 | (-0.01 to 0.03) | 0.04 | | 0.01 | (-0.02 to 0.04) |

Relative age effects on multiple domains, adjusted for actual age at testing and gender (main effects only), and models including an interaction of relative age effects by gender, for adolescents with a normative school progress. Regression estimates were bootstrapped (*k*=10,000 with bias corrected intervals), and indicate change in outcome per month in relative age, after adjustment for age at testing and gender. Note that for change variables we also adjusted for change in age between *T*_1_ and *T*_3_. Interaction effects were tested in a model with all main effects.

*Note*. *n*= 1681. Gender: female= 0; ∆= change between *T*_1_ (age 11) and *T*_3_ (Age 16); BMI= body mass index; All models explained 0.5 to 1.5% of the variance, and change models 2%. Details on all measures and procedures can be found in the method section. All correlations between all variables are given in Table 2. Significance: ^***^*p*<.001, ^**^*p*<.01, ^*^*p*<.05, two-tailed.

†= A robustness test of relative-age effects on length, adjusted for biological age and gender, was not significant in our alternative relative-age sample (*n*= 794, *β*= -0.04, *p*= .33, B= -0.10, *p*= .35, 95%CI= -0.30 to 0.11), and neither was the interaction effect in the second model (*β*= 0.09, *p*= .40, B= 0.16, *p*= .42, 95%CI= -0.23 to 0.55). This suggests that the effect in the table is an artifact of the correlation between relative age and biological age, which may be larger in boys than girls because their puberty takes off later.
