# Supplementary material for: Relative Age Effects in Dutch Adolescents: Concurrent and Prospective Analyses
Source: PLoS One. 2015 Jun 15;10(6):e0128856. doi: 10.1371/journal.pone.0128856 (PMC4468064; doi:10.1371/journal.pone.0128856)
Supplement: S9 Table — (DOCX) [file pone.0128856.s009.docx]

**S9 Table.**

Percentage of children with a specific relative age position by socioeconomic status

|  | **Repeated a grade** | | | | | | **Skipped a grade** | | | | **Special education** | | | |
| --- | --- | --- | --- | --- | --- | --- | --- | --- | --- | --- | --- | --- | --- | --- |
| **RA** | **Low SES** | | | **High SES** | | | **Low SES** | | **High SES** | | **Low SES** | | **High SES** | |
|  | ***n*** | **%** |  | ***n*** | **%** |  | ***n*** | **%** | ***n*** | **%** | ***n*** | **%** | ***n*** | **%** |
| 1. | 16 | 12.9% |  | 13 | 27.7% |  |  |  |  |  | 2 | 3.3% | 1 | 25.0% |
| 2. | 25 | 20.2% |  | 13 | 27.7% |  |  |  |  |  | 5 | 8.2% | 1 | 25.0% |
| 3. | 17 | 13.7% | 46.8% | 3 | 6.4% | 61.7% |  |  | 1 | 5.9% | 5 | 8.2% | 2 | 50.0% |
| 4. | 9 | 7.3% |  | 3 | 6.4% |  |  |  |  |  | 4 | 6.6% |  |  |
| 5. | 13 | 10.5% |  | 1 | 2.1% |  |  |  | 1 | 5.9% | 5 | 8.2% |  |  |
| 6. | 9 | 7.3% | 71.8% | 2 | 4.3% | 74.5% | 1 | 20.0% |  |  | 10 | 16.4% |  |  |
| 7. | 12 | 9.7% |  | 3 | 6.4% |  |  |  | 2 | 11.8% | 3 | 4.9% |  |  |
| 8. | 6 | 4.8% |  | 5 | 10.6% |  |  |  |  |  | 6 | 9.8% |  |  |
| 9. | 4 | 3.2% | 89.5% | 3 | 6.4% | 97.9% | 1 | 20.0% |  |  | 6 | 9.8% |  |  |
| 10. | 7 | 5.6% |  |  |  |  | 1 | 20.0% | 3 | 17.6% | 3 | 4.9% |  |  |
| 11. | 4 | 3.2% |  |  |  |  | 2 | 40.0% | 3 | 17.6% | 9 | 14.8% |  |  |
| 12. | 2 | 1.6% |  | 1 | 2.1% |  |  |  | 4 | 23.5% | 3 | 4.9% |  |  |
|  | 124 | 100% |  | 47 | 100% |  | 5 | 100% | 17 | 100% | 61 | 100% | 4 | 100% |

Relative age (RA) in months for adolescents who (a) repeated a grade, (b) skipped a grade, or (c) went to special education, for lowest and highest quartile of socioeconomic status (SES)
